# *Varestrongylus eleguneniensis* sp. n. (Nematoda: Protostrongylidae): a widespread, multi-host lungworm of wild North American ungulates, with an emended diagnosis for the genus and explorations of biogeography

**DOI:** 10.1186/s13071-014-0556-9

**Published:** 2014-12-17

**Authors:** Guilherme G Verocai, Susan J Kutz, Manon Simard, Eric P Hoberg

**Affiliations:** Department of Ecosystem and Public Health, Faculty of Veterinary Medicine, University of Calgary, 3280 Hospital Drive NW, Calgary, Alberta T2N 4Z6 Canada; Canadian Wildlife Health Cooperative - Alberta Node, Faculty of Veterinary Medicine, University of Calgary, 3330 Hospital Drive NW, Calgary, Alberta T2N 4N1 Canada; Nunavik Research Centre, Makivik Corporation, Kuujjuaq, Quebec J0M 1C0 Canada; Current address: 936 rue des Prairies, apt 213, Québec, G1K 8T2 Canada; United States National Parasite Collection, US Department of Agriculture, Agricultural Research Service, BARC East No. 1180, 10300 Baltimore Avenue, Beltsville, Maryland 20705 USA

**Keywords:** *Alces americanus*, Cervidae, Nearctic, *Rangifer tarandus*, *Ovibos moschatus*, Taxonomy, Varestrongylinae, Verminous pneumonia

## Abstract

**Background:**

A putative new species of *Varestrongylus* has been recently recognized in wild North American ungulates based on the ITS-2 sequences of larvae isolated from feces during a wide geographic survey. No taxonomic description was provided, as adult specimens were not examined.

**Methods:**

Lungworm specimens were collected in the terminal bronchioles of muskoxen from Quebec, and a woodland caribou from central Alberta, Canada. The L3 stage was recovered from experimentally infected slugs (*Deroceras* spp.). Description of specimens was based on comparative morphology and integrated approaches. Molecular identity was determined by PCR and sequencing of the ITS-2 region of the nuclear ribosomal DNA, and compared to other protostrongylids.

**Results:**

*Varestrongylus eleguneniensis* sp. n. is established for a recently discovered protostrongylid nematode found in caribou (*Rangifer tarandus*), muskoxen (*Ovibos moschatus*) and moose (*Alces americanus*); hosts that collectively occupy an extensive geographic range across northern North America. Adults of *Varestrongylus eleguneniensis* are distinguished from congeners by a combination of characters in males (distally bifurcate gubernaculum, relatively short equal spicules not split distally, a strongly elongate and bifurcate dorsal ray, and an undivided copulatory bursa) and females (reduced provagina with hood-like fold extending ventrally across prominent genital protuberance). Third-stage larvae resemble those found among other species in the genus. The genus *Varestrongylus* is emended to account for the structure of the dorsal ray characteristic of *V. eleguneniensis*, *V. alpenae*, *V. alces* and *V. longispiculatus*.

**Conclusions:**

Herein we describe and name *V. eleguneniensis*, a pulmonary protostrongylid with *Rangifer tarandus* as a primary definitive host, and which secondarily infects muskoxen and moose in areas of sympatry. Biogeographic history for *V. eleguneniensis* and *V. alpenae,* the only two endemic species of *Varestrongylus* known from North America, appears consistent with independent events of geographic expansion with cervid hosts from Eurasia into North America during the late Pliocene and Quaternary.

## Background

Nematodes of the Family Protostrongylidae Leiper, 1926 are characteristic and often pathogenic parasites among Bovidae, particularly Caprinae, and Cervidae (Artiodactyla) across the Holarctic (comprised by the Palaearctic and Nearctic regions) [1-3], and less frequently in tropical regions of the southern hemisphere [4,5]. The Nearctic protostrongylid fauna includes genera and species, partitioned among four subfamilies, occurring in domestic and free-ranging ungulates and lagomorphs: Protostrongylinae Kamensky, 1905; Muelleriinae Skrjabin, 1933; Elaphostrongylinae Boev & Shulz, 1950; and Varestrongylinae Boev, 1968, e.g. [2]. Species within the Protostrongylinae, Muelleriinae, and Varestrongylinae are strictly pulmonary parasites, with adult nematodes residing in the bronchi, bronchioles, or lung parenchyma, whereas those of the Elaphostrongylinae are found in the skeletal musculature or central nervous system of their hosts. Although the protostrongylid assemblage of Nearctic ungulates was thought to be well defined, there is a growing body of knowledge regarding their biodiversity with new insights about historical processes, host range, geographic distribution and faunal structure, e.g. [6-15].

Within the Varestrongylinae, the genus *Varestrongylus* Bhalerao, 1932 is of special interest due to its complex taxonomic history [1,16,17]. Currently, there are nine nominal species considered valid within the genus, with free-ranging cervids and wild and domestic caprine bovids as the main hosts [1,18-20]. The center of diversity for the genus is Eurasia [13], where eight species have been described.

Until recently, *Varestrongylus alpenae* (Dikmans, 1935) a lungworm in the white-tailed deer, *Odocoileus virginianus* (Zimmermann), occurring at temperate and subtropical regions of southern, eastern and central North America [12,21-26], was recognized as the sole species of *Varestrongylus* endemic in North America. However, during the last decade molecular-based survey of high latitude protostrongylids in North America resulted in the detection of a putative second Nearctic species of *Varestrongylus* [12]. This parasite was distinguished from other protostrongylids based on sequences of the internal transcribed spacer region-2 (ITS-2) of the nuclear ribosomal DNA (rDNA) derived from first-stage dorsal-spined larvae (DSL) extracted from ungulates feces [12]. Although sequence data suggested its placement within the genus *Varestrongylus*, considerable divergence was demonstrated relative to *V. alpenae* see [12].

At the time of the original discovery [12], field collections indicated a broad geographic range, extending throughout the northern Nearctic, encompassing five Canadian provinces and territories: mainland Labrador and Newfoundland, Quebec (QC), Nunavut (NU), Northwest Territories and the Yukon, as well as Alaska, USA [12]. More recently, this undescribed species was found on Victoria Island in the Canadian Arctic, demonstrating that its distribution is not restricted to mainland North America [15]. The host range of this new species is remarkably broad, with natural infections detected in caribou of three subspecies: the woodland caribou, *Rangifer tarandus caribou* (Gmelin), the barrenground caribou, *Rangifer tarandus groenlandicus* (Borowski), and the Grant’s caribou, *Rangifer tarandus granti*; muskoxen of two subspecies, *Ovibos moschatus moschatus* (Zimmermann) and *Ovibos moschatus wardi* Lyddeker; and one subspecies of moose, *A. americanus gigas* Miller [12,15,27]; Verocai, Kutz, unpublished data.

Protostrongylids have an indirect life-cycle, requiring gastropods as intermediate hosts (IH) [1,3,28]. To date, the only known naturally infected IH is the meadow slug *Deroceras laeve* (Müller, 1774) [12]. This species is widely distributed across northern North America [29] and is believed to be the main intermediate host for several protostrongylids [27].

Recent collections of adult nematodes from the bronchioles of caribou and muskoxen now allow a complete description and series of comparisons to characterize this previously unknown species. In this study we propose the establishment of *Varestrongylus eleguneniensis* sp. n. for this geographically wide-spread, multi-host protostrongylid lungworm occurring across northern North America. Further, we provide an emended diagnosis for the genus *Varestrongylus*, and explore the historical biogeography of these nematodes in the Nearctic.

## Methods

### Taxonomic criteria

Host taxonomic classification follows Grubb [30] and Hernández-Fernández and Vrba [31]. Parasite taxonomy is largely consistent with the latest revision [1] and the most recent phylogenetic hypothesis for the family Protostrongylidae [2].

### Collection

*Muskoxen*: Muskoxen were harvested from a free-ranging population in Nunavik, QC. These animals were introduced in 1967 from Ellesmere Island, NU to Old Fort Chimo located across the Koksoak River, near Kuujjuaq, for farming purposes, mainly for qiviut production (muskox wool). The farm was shut down and animals were released between 1973 and 1983, and their descendants are currently distributed throughout much of the Ungava Peninsula in northern QC extending eastwards into Labrador [32,33]. All muskoxen examined in this study were harvested through either subsistence or sport hunting regulated by the Ministry of Natural Resources and Fauna of Quebec (Ministère des Ressources Naturelles et de la Faune du Québec). Hunters submitted selected samples of harvested muskoxen to the Nunavik Research Centre (NRC), Makivik Corporation, located in Kuujjuaq, for general health and food safety assessment (zoonotic diseases and contaminants). Nematodes were isolated from the lungs of two muskoxen harvested near the town of Tasiujaq, QC in late March, 2010: an adult female (Om-01-2010, March 20th, 58°44′51″N, 70°02′06″W) and an adult male (Om-02-2010, March 28th, 58°44′10″N, 69°34′18″W). Another adult female (Om-10-2010) found dead on 31 December, 2009 near Kuujjuaq (58°06′24″N, 68°23′55″W) was collected by NRC personal and kept frozen until necropsy. Additionally, a single female nematode, collected from an adult male muskox (Om-10-2007) hunted near Kuujjuaq (58°45′00″N, 68°33′29″W) on March 21st, 2007, was preserved in 70% ethanol by M. Simard, and later identified as *Varestrongylus*, and also used for the species description.

#### Caribou

Additional adult nematodes were collected from the lungs of an adult male woodland caribou belonging to the Cold Lake herd, Alberta, Canada. The animal was found dead, likely killed by collision with motor vehicle, at 55°2′25″N, 110°34′5″W, near the border with Saskatchewan. The carcass was collected by the Fish and Wildlife Division (FWD) of the Alberta Sustainable Resource Development under the Alberta Wildlife Research Permit no. 48549.

### Fecal analyses

Fecal samples of the three muskoxen and the woodland caribou were collected and kept frozen at −20°C until analyses. Samples were analyzed for the presence of protostrongylid DSL using the modified beaker Baermann technique [34] at the NRC and the University of Calgary, prior to lung dissection.

### Lung dissection

Lungs were thawed and individually processed. Briefly, they were first grossly examined for pathology and presence of nematodes. A first wash was done with the lungs still intact by flushing tap water into the trachea and then pouring the fluid from the lungs back through a 75 μm mesh sieve. Material retained on the sieve was put in Petri dishes and examined under a stereomicroscope for the presence of nematodes. The entire bronchial tree was then dissected with repeated washing of the exposed airways and pulmonary tissue through the sieve. Material retained in the sieve was examined as described above. All intact nematodes or fragments were collected, identified by gender, and stored in 70% ethanol.

### Morphological identification

#### Nematodes examined

*Adults*. Specimens and fragments of adult nematodes were mounted and cleared in either lactophenol or phenol-alcohol, and examined under light microscopy with differential interference optics. Photomicrographs were prepared with a Nikon DX 1200 digital camera and a Zeiss Axiophot microscope. Line drawings were prepared with the use of a drawing tube. Throughout the descriptions, measurements are given in micrometres (μm) unless specified otherwise, and are presented with the numbers of adult male, female or larval (DSL and third-stage larvae, L3) nematodes examined (n=), and the range is followed by the mean ± 1 SD within parentheses. *First-stage larvae.* DSL from feces were recovered from muskox Om-02-10 using the modified beaker Baermann technique [34]. Isolated live DSL were killed in steaming 70% ethanol/glycerine solution (19:1), and held for further evaluation. Species identity was confirmed by molecular sequencing prior to the use of DSL to establish experimental infections in slug intermediate hosts (see below). *Third-stage larvae.* DSL for infection of slugs *D. laeve* and *Deroceras reticulatum* were isolated from feces of the same muskox population at Nunavik, QC (UC-133, representative DSL ITS-2 sequence in Table 1). The slugs were experimentally exposed to 300 DSL/slug as per [35]. Gastropods were maintained in Petri dishes for a day and transferred to plastic containers with an autoclaved soil/vermiculite mix kept at 20°C, making sure that there was constant moisture, and provided with food (lettuce and carrot). Slugs were killed between 18–21 or 50–60 days post exposure (*D. laeve* and *D. reticulatum*, respectively), cut into small pieces, and digested in HCl/pepsin solution [6,36]. Material was analyzed under a dissecting microscope, and all L3 recovered were preserved in 70% ethanol.

**Table 1:** **Summary of collected specimens of** ***Varestrongylus eleguneniensis*** **sp. n. from muskoxen (** ***Ovibos moschatus wardi*** **) from Nunavik Region, Quebec, Canada, and woodland caribou (** ***Rangifer tarandus caribou*** **) from Alberta, Canada**

**Animal ID**	**Species**	**Sex**	**LPG** ^**a**^	**Coordinates**	**Locality**	**Number of adult specimens**				**Accession Numbers**	
						**Males**	**Male fragments**	**Females**	**Female fragments**	**USNPC**	**GenBank (ITS-2)**
Om-10-07	*O. m. wardi*	Male	-	58° 45′00″N 68° 33′29″ W	Kuujjuaq, QC	0	0	1	0	103743 (♀)	–
Om-01-10	*O. m. wardi*	Female	6	58° 44′51″N 70° 02′06″W	Tasiujaq, QC	1*	0	0	0	103740 (♂)*	JQ478746
Om-02-10	*O. m. wardi*	Male	25	58° 44′10″N 69° 34′18″W	Tasiujaq, QC	3	1 tail	1**	1 head, 3 tails	103741 (♀)** 103742 (♂,♀)^**†**^ 103748 (♀) 103749 (♀)	– JQ478649 (♂) JQ478647 JQ478648
Om-10-10	*O. m. wardi*	Female	0.4	58°45′00″N 68° 33′29″W	Kuujjuaq, QC	4	1 tail	2	1 tail	103744 (♀,♂)^**†**^	JQ478644 (♀) JQ478645 (♂)
UC178-2	*R. t. caribou*	Male	0.4	55° 2′25″N 110° 34′5″W	Cold Lake, AB	1^§^	1 head, 1 tail	0	0	105697 (♂) 105698 (♂) 105699 (♂) 105700 (♂)^§^ DSL^§§^	JX115007 − JX115007 − JQ478651^§§^

All these specimens were used for the taxonomical description (holotype, allotype and paratypes), with matching accession numbers at the United States National Parasite collection (USNPC) and for sequences at the second internal transcript spacer (ITS-2) region at the nuclear ribosomal DNA deposited at GenBank.

^a^Protostrongylid first-stage larvae (DSL) per gram of feces; *Holotype; **Allotype; ^†^Multiple vials containing males and females (intact specimens or fragments), USNPC103742 also contains a vial with DSL; ^§^Additional immature male not included in the description, but accessioned at the USNPC as a voucher; ^§§^Additional sequence from DSL extracted from host feces, not accessioned at USNPC.

### Molecular analyses

#### DNA extraction and amplification

Adult nematode fragments were recovered and subsampled from each of the three muskoxen and the caribou. These fragments were individually transferred into wells prior to DNA extraction. Of these, eight fragments (6 from the 3 muskoxen, and 2 from the caribou) had matching caudal extremities or caudal and cephalic extremities used in the morphological description of the species, and, therefore, are part of the type-series (holotype and paratypes) deposited in the United States National Parasite Collection (USNPC), Agricultural Research Service, USDA, Beltsville, MD, USA (see Table 1). In accordance with section 8.5 of the ICZN’s International Code of Zoological Nomenclature, details of the new species have been submitted to the ZooBank under the life science identifier (LSID) zoobank.org:pub:0E9BC9BC-EE4F-461E-9000-37FCFEB4C71F.

Genomic DNA (gDNA) was extracted from 2–4 mm nematode fragments in 2 mL tubes containing 5 μL of deionized water. To each tube was added 25 μL of lysis buffer (0.5 mg/mL of proteinase K, 10× PCR buffer). DNA extraction followed the following protocol: tubes containing adult worm fragments were incubated at 60°C for 60 min, 65°C for 60 min, then at 95°C for 15 min. The lysis was repeated, after the addition of 1 μL of proteinase K (20 mg/mL) in each well, following the same protocol. Extracted DNA was diluted 1:10. For species identification, a PCR modified from [12] was performed using primers NC1 (5′-ACG TCT GGT TCA GGG TTG TT-3′) and NC2 (5′- TTA GTT TCT TTT CCT CCG CT-3′) targeting the ITS-2 region of rDNA. PCR amplification was performed in 40 μL reactions containing: 20.4 μL of water, 8 μL of 10× PCR buffer + MgCl_2_, 0.8 μL of 10 mmol dNTPs, 4 μL (10 μM) of each primer, 0.4 μL of bovine serum albumin, 0.4 μL of *Taq* Phusion HF DNA polymerase, and 2 μL of DNA template. The amplification conditions used were an initial 2 min denaturation at 98°C, followed by 35 cycles of 98°C for 10 s, 52.5°C for 30 s, and 72°C for 30 s, annealing. A final extension phase of 72°C for 5 min was followed by cooling to 10°C.

PCR products were cleaned using ExoSAP-it® and sequenced directly using NC1 and NC2 primers using BigDye Terminator Cycle Sequencing (Applied Biosystems).

#### Molecular identification of larvae

Specific identity of first-stage larvae used for gastropod infection (L3 description) and from the woodland caribou (UC178-2) was also based on PCR and sequencing of the ITS-2 region of the nuclear ribosomal DNA. DNA lysis was performed as described above, and PCR was done using the same NC1 and NC2 primers. Each in 20 μL reactions contained 10.2 μL of water, 4 μL of 5× PCR buffer + Mg, 0.4 μL of 10 mmol dNTPs, 2 μL (10 μM) of each primer, 0.2 μL of *Taq* Phusion HF DNA polymerase, 0.2 μL of bovine serum albumine (20 mg/mL), and 1 μL of DNA template. The amplification conditions used were the same as described above. PCR products were also cleaned and sequenced as previously described, and sequences analyzed accordingly. Representative sequences were deposited in GenBank.

#### Sequence analyses

Direct sequences at the ITS-2 locus of adults and larvae of *V. eleguneniensis* produced in the present study were edited using FinchTV 1.4.0 (Geospiza Inc.) and MEGA version 5 [37]. ITS-2 sequences were compared with those of putative *V. eleguneniensis* sequences [12,15], and those for other protostrongylids from eight genera and 13 species represented in the original finding of this new species [12] and available from GenBank: *Varestrongylus alpenae* (AY648407), *Parelaphostrongylus andersoni* (AF504030), *Parelaphostrongylus odocoilei* (AF504031), *Parelaphostrongylus tenuis* (Dougherty, 1945) (AF504029), *Elaphostrongylus rangiferi* (Mitskevitch, 1960) (EU018482, AF504033), *Elaphostrongylus alces* Stéen, Chabaud & Rehbinder, 1989 (AF504034), *Elaphostrongylus cervi* Cameron, 1931 (AF504026), *Umingmakstrongylus pallikuukensis* Hoberg, Polley, Gunn & Nishi, 1995 (AY648409), *Muellerius capillaris* (Mueller, 1889) (AY679527), *Cystocaulus ocreatus* (Railliet & Henry, 1908) (EU018481), *Orthostrongylus macrotis* (Dikmans, 1931) (EU018483), *Protostrongylus stilesi* Dikmans, 1931 (EU018484), and *Protostrongylus rufescens* (Leuckart, 1965) (EU018485). Sequences were then aligned using PRANK, a probabilistic multiple alignment program available through the European Bioinformatics Institute (http://www.ebi.ac.uk/goldman-srv/prank).

The maximum identity of the *V. eleguneniensis* ITS-2 sequences (including range, average and SD) obtained in this study and those previously produced for this species [12,15] was calculated using the pairwise distance matrix produced by Geneious [38]. In order to test the hypothesis of conspecificity of isolates, including the holotype and paratypes for *V. eleguneniensis*, and selected sequences from [12] (n = 8) and [15] (n = 2), an unrooted neighbour-joining tree was designed in Geneious [38], using the substitution model HKY, with gaps treated as complete deletion, and 5,000 bootstrap replicates, including also the sequences of all protostrongylid species mentioned above.

### Other specimens examined

Specimens of *Varestrongylus alces* Demidova & Naumitscheva, 1953, *V. alpenae*, *Varestrongylus pneumonicus* Bhalerao, 1932, *Varestrongylus sagittatus* (Mueller, 1890), and specimens attributable to *Varestrongylus capreoli* (Stroh & Schmid, 1938) (referred as *Varestrongylus* cf. *capreoli* in [20]) available in the USNPC were examined and compared to those of the undescribed species during the development of the morphological description (see Table one from [20]). Representative specimens of other species were not immediately available for comparison.

### Community consultation

The first DSL confirmed as belonging to this novel protostrongylid species were isolated from feces of a barrenground caribou belonging to the Bluenose East herd that ranges in the Sahtu Settlement Area (SSA), NT [12] and vicinities. Therefore, we returned to three communities in the SSA: Deline (Délînê), Fort Good Hope (Rádeyîlîkóé), and Colville Lake (K’áhbamį́túé), where Sahtu Dene elders and hunters were consulted on naming the parasite species in their language. The name proposed was based on the North Slavey (Sahtúot’ı̨nę Yatį́) language, which belongs to the northwestern Canada group of the Northern Athabaskan language family [39].

## Results

### Lung and fecal analyses, and specimens examined

No gross pulmonary lesions indicative of parasite infection were observed in the three muskoxen and one caribou. Nine entire male and four entire female nematodes and four male fragments and five female fragments containing relevant morphological characteristics were recovered from four muskoxen and the woodland caribou (Table 1). Also, numerous DSL and eggs containing different developmental stages (from morula to fully developed DSL) were found in lung washes. Results for larvae per gram (LPG) of feces for the muskoxen and the woodland caribou are provided in Table 1.

### Molecular findings

**Figure 1:**
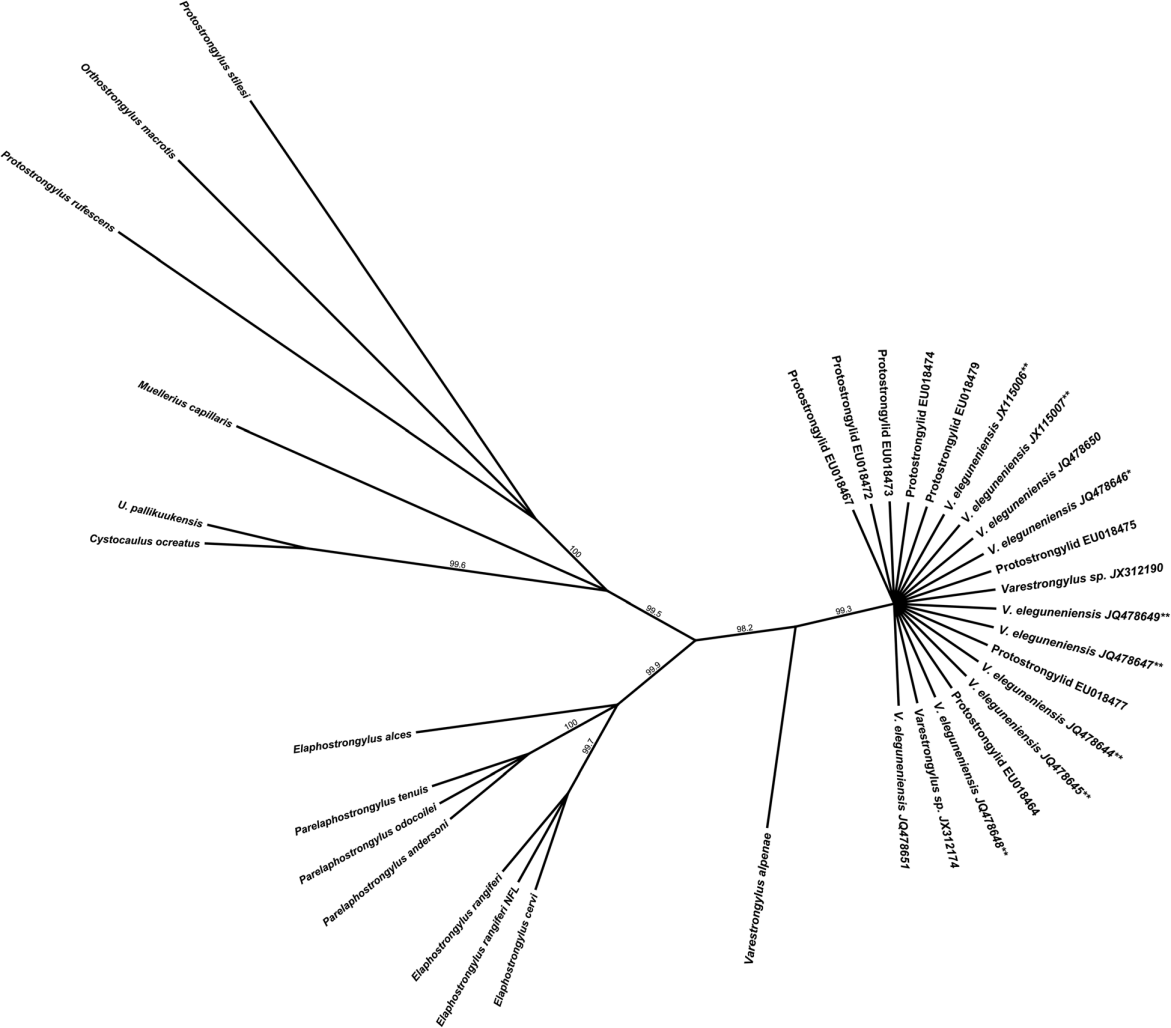
**Unrooted neighbor-joining tree of the ITS-2 region, demonstrating reciprocal monophyly of** ***Varestrongylus eleguneniensis*** **.** Unrooted neighbor-joining tree based on HKY distances at the ITS-2 region of the nuclear ribosomal DNA, demonstrating the identity of adult and larval specimens of *V. eleguneniensis* produced in the current study and those previously attributed to an unknown species of *Varestrongylus* at high altitudes of North America. Selected sequences shown in this tree represent adults, L1 and L3 of *V. eleguneniensis* from the current study, L1 ‘Protostrongylid’ from [12] and L1 of ‘*Varestrongylus* sp.’ from [15]. Sequences at the ITS-2 locus for other genera and species of protostrongylids included those used in the original comparisons by [12]; GenBank accession numbers in Methods). Bootstraps (5,000) values are only shown for branches with over 95% support. Superscript (*) refers to the holotype and (**) to paratypes of *V. eleguneniensis* deposited at the United States National Parasite Collection (see Table 1).

Genetic identity (similarity) based on ITS-2 sequences from 10 adult nematodes from this study and DSL belonging to the putative species of *Varestrongylus* discovered and reported by Kutz et al. [12,15] was 95–100% (98.7 ± 1.4). The neighbor-joining tree supports the conspecificity of these widespread populations of *Varestrongylus* represented by adults and larval parasites (Figure 1). Further, reciprocal monophyly is demonstrated relative to the putative sister species within *Varestrongylus* (99.3 bootstrap support), and homologous sequences are distinct among other species of *Varestrongylus* where molecular data are available (see [20]; present study). The independent nature of *Varestrongylus* among related protostongylids and within the subfamilies Elaphostrongylinae, Muelleriinae, and Protostrongylinae is confirmed. Sequences from adult nematodes were deposited in the GenBank under accession numbers: JQ478644–49 for muskox isolates and for JX115006–07 for caribou isolates; all of these are linked to vouchers in the type series in the USNPC (see Table 1). Representative ITS-2 sequences were deposited for DSL used for gastropod infection and from the same caribou (JQ478650 and JQ478651, respectively).

### Description

#### *Varestrongylus eleguneniensis***sp. n.**

**Figure 2:**
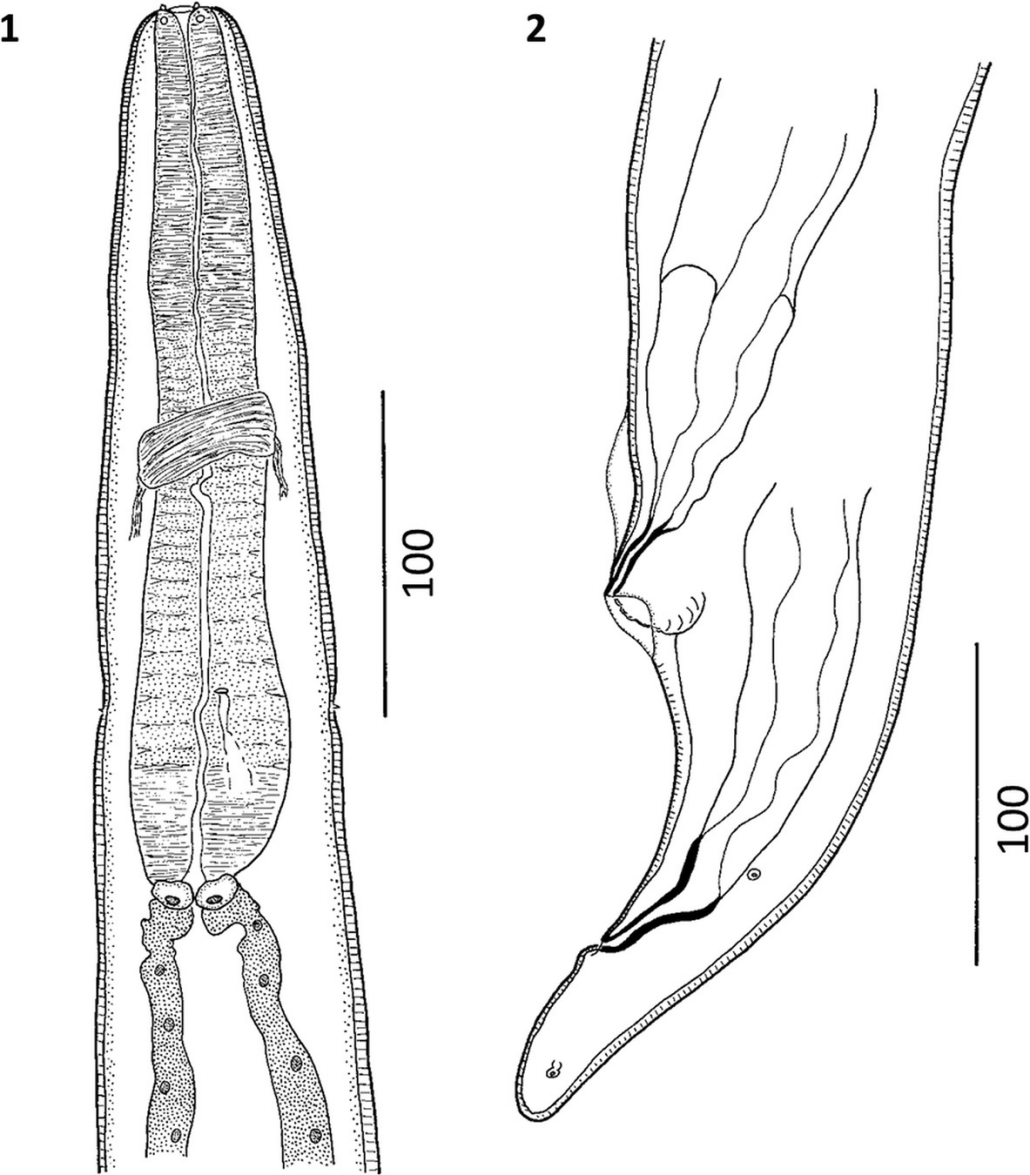
***Varestrongylus eleguneniensis*** **sp. n. female. 2**. Cephalic extremity at ventral view. **3**. Caudal extremity at lateral view, note poorly developed provagina.

**Figure 3:**
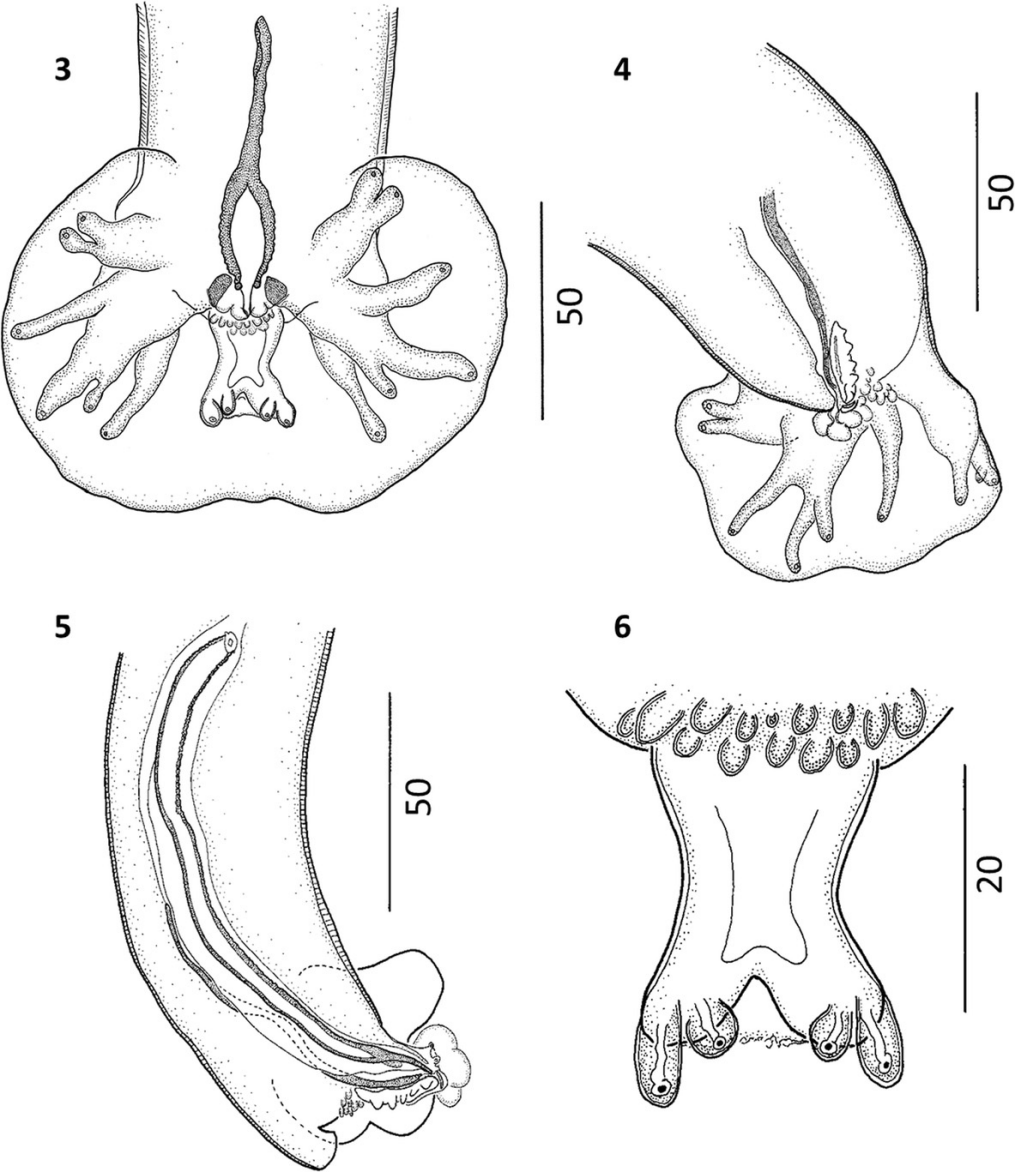
***Varestrongylus eleguneniensis*** **sp. n. male, caudal extremity. 4**. Ventral view, note the dorsally notched copulatory bursa and the disposition of bursal rays, and bifurcate gubernaculum. **5**. Ventro-lateral view, note the denticulate plate of crura, and genital protuberances. **6**. Lateral view: spicule, partially covering gubernaculum, denticulate plates of crura. **7**. Ventral view, detail on the elongate, bifurcate dorsal ray, and genital protuberances.

**Figure 4:**
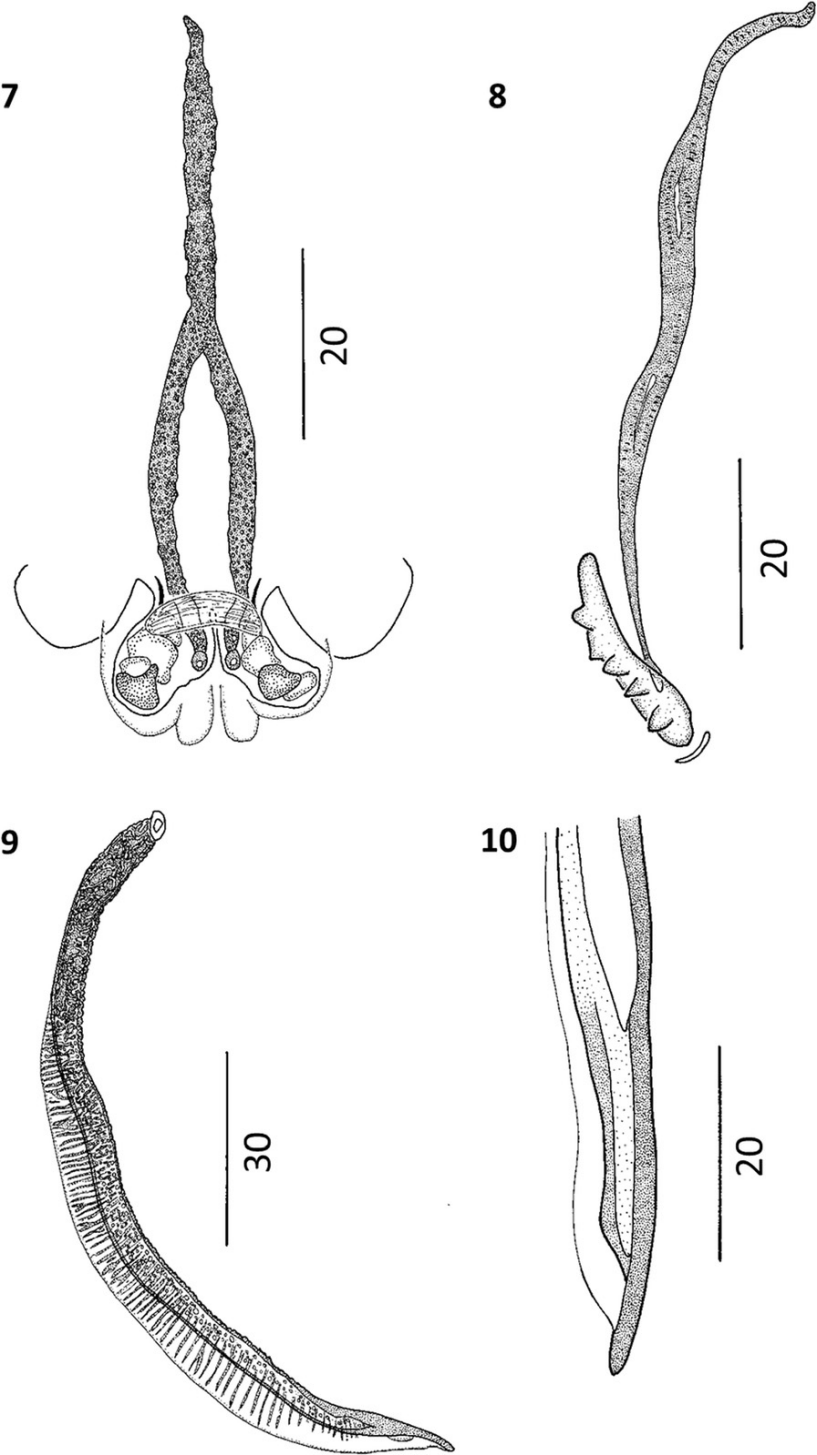
***Varestrongylus eleguneniensis*** **sp. n. male. 8**. Ventral view of gubernaculum and denticulate plates of crura. **9**. Lateral view of gubernaculum and denticulate plates of crura, and poorly developed telamon plate. **10**. Lateral view of spicule; **11**. Lateral view of spicule tip, non-split and ending in a finger-like projection.

**Figure 5:**
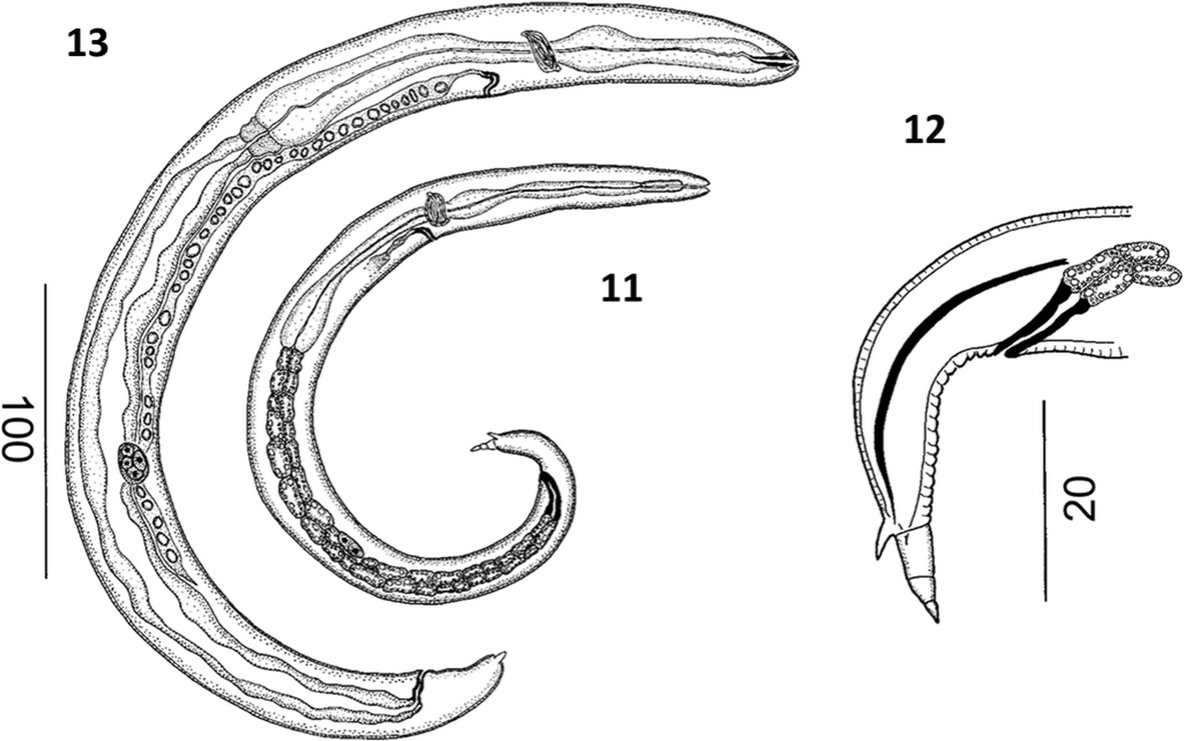
***Varestrongylus eleguneniensis*** **sp. n. larval stages. 12**. First-stage larva (L1 or dorsal spined-larva, DSL) at lateral view. **13**. Detail on caudal extremity of the First-stage larva (dorsal spined-larva) at lateral view. **14**. Third-stage larva (L3) at lateral view.

**Figure 6:**
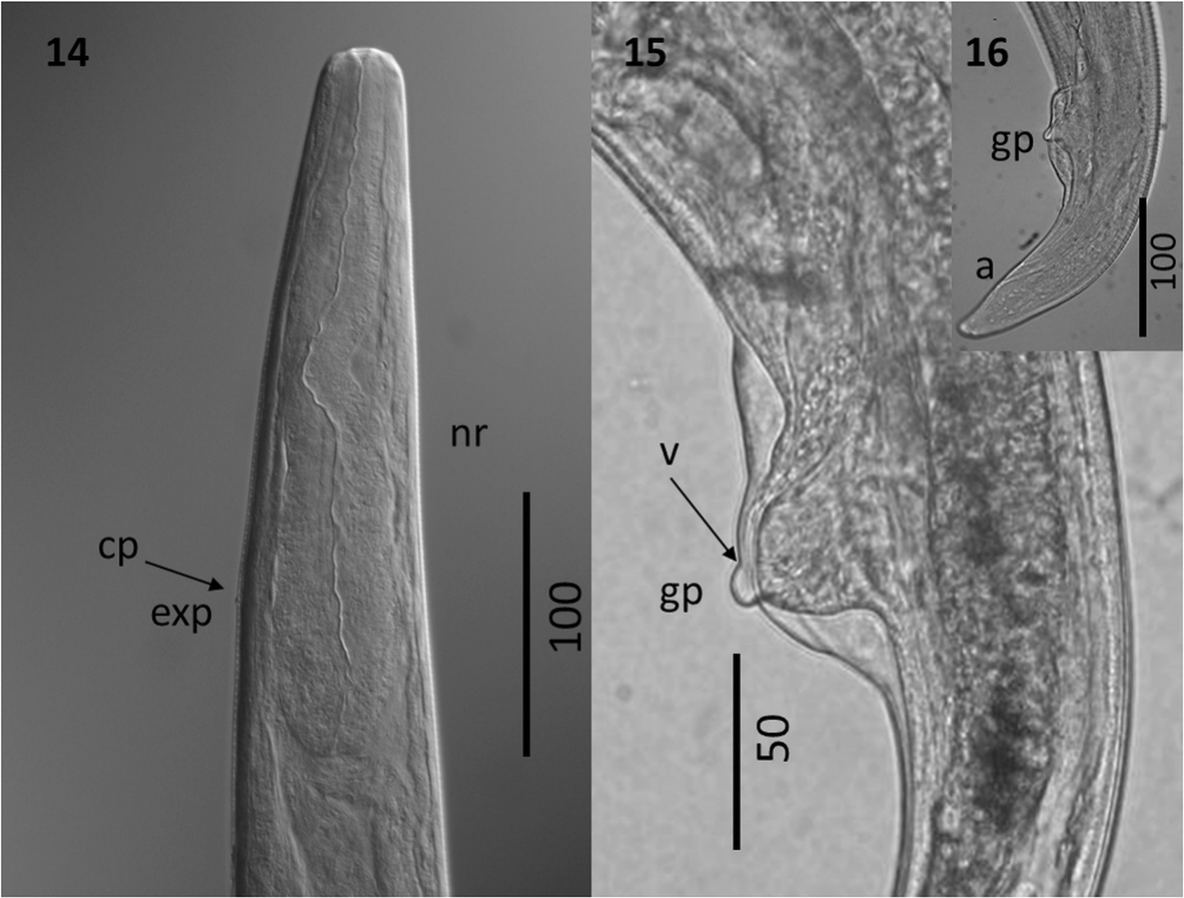
***Varestrongylus eleguneniensis*** **sp. n. female. 15**. Cephalic extremity of a female specimen at ventral view, note the claviform esophagus, nerve-ring (nr), and cervical papillae (cp), excretory pore (ep) located at posterior third of esophagus (40×).**16**. Caudal extremity of a female specimen at lateral view, note the poorly developed provagina, genital protuberance (gp) and vaginal opening (v), (40×).**17**. Caudal extremity of a female specimen at lateral view, showing the anus (a), and the conical tail (40×).

**Figure 7:**
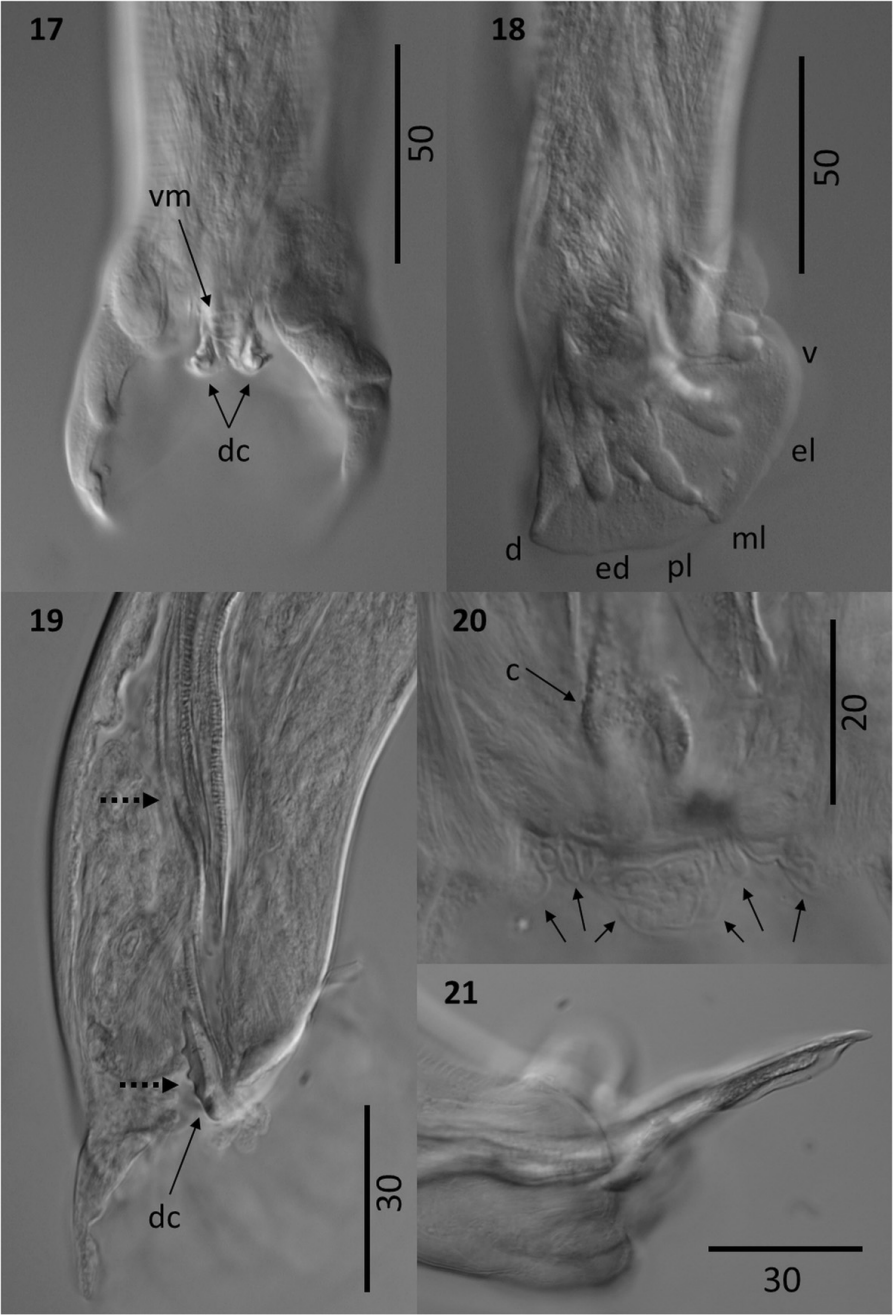
***Varestrongylus eleguneniensis*** **sp. n. male. 18**. Caudal extremity ventral view: cuticular striations, denticulate plates of crura (dc), (40×). **19**. Caudal extremity at lateral view showing disposition of the bursal rays: ventral (v), externo-lateral (el), medio-lateral (ml), postero-lateral (pl), externo-dorsal (ed), dorsal (d) (40×). **20**. Caudal extremity at lateral view: distal portion of spicule and gubernaculum, and gubernaculum (dashed arrows) and denticulate plates of crura (dc), (100×). **21**. Detail on caudal extremity of a male specimen: crura (c), and genital protuberances (arrows) (160×). **22**. Lateral view of the tip of protruded spicule, non-split and ending in a finger-like projection (100×).

**Figure 8:**
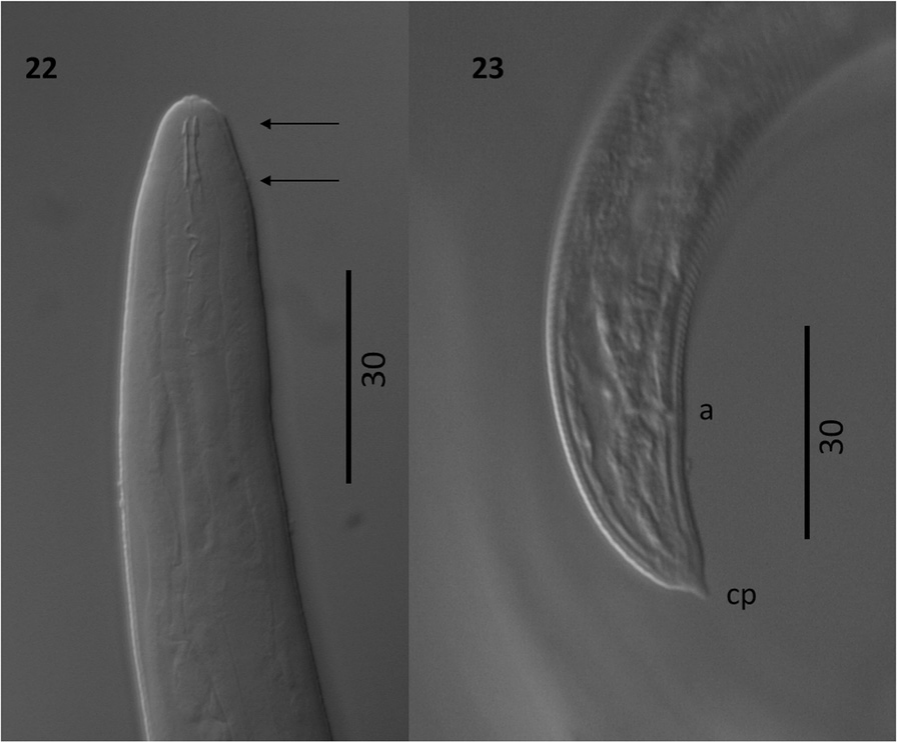
***Varestrongylus eleguneniensis*** **sp. n. third-stage larva (L3). 23**. Cephalic extremity of L3 at dorsal view: buccal structures, stylets (arrows), and anterior part of esophagus (100×). **24**. Caudal extremity of L3 at lateral view: detail on the cuticular striations, anus (a), and tail spike (ts) or caudal protuberance (64×).

**General description:** (Figures 2, 3, 4, 5, 6, 7 and 8). Protostrongylidae, Varestrongylinae, minuscule, thread-like nematodes, reddish-brown prior to fixation, with delicate cuticle marked by transverse striations. Cephalic extremity bluntly rounded. Buccal aperture surrounded by four papillae. Esophagus cylindrical, clavate, broadening at base, poorly demarcated into muscular and glandular regions. Nervering indistinct, located in anterior third or mid-third of esophagus; minuscule cervical papillae and excretory pore usually situated posterior to nerve ring, in middle or posterior third of esophagus.

**Males:** Based on specimens in muskoxen (8 intact males, including holotype, and two posterior end fragments) and caribou (one intact male, one anterior end, and one posterior end). Total length (n = 10) 8.8–14.7 mm (11.2 ± 1.64); maximum width (n = 10) 78–147 (102.7 ± 18.08) attained approximately at mid-body. Esophagus (n = 9) 247–395 (325.7 ± 51.23) long, 44–62 (50.9 ± 6.35) wide, (n = 8) 2.8–3.5% (2.9 ± 0.46%) of body length. Nerve-ring (n = 7) 87–196 (142.1 ± 37.11), cervical papillae (n = 4) 151–215 (187.5 ± 27.43), excretory pore (n = 7) 147–242 (202.3 ± 37.27) from cephalic extremity. Copulatory bursa rounded, lacking distinct lobes, slightly notched at posterior margin. Bursal rays approaching, but rarely attaining margin of bursa. Body width at bursa (n = 12) 50–75 (59.2 ± 6.90), bursa length (n = 11) 65–91 (79.5 ± 7.27), bursa width (n = 11) 95–135 (110.2 ± 9.77). Ventroventral and lateroventral rays equal, arising from common stalk, parallel to one another, tips of rays distally separate, and directed anteriad and isolated from others. Lateral rays arising from common base; externo-lateral elongate, reaching margin and isolated from medio- and postero-lateral rays. Medio-lateral ray long and postero-lateral ray reduced, tips separate from near middle or less than half of common stalk. Externo-dorsal rays long, origins independent from base of dorsal ray. Dorsal ray elongate (n = 9) 23–39 (29.2 ± 6.10) long, (n = 8) 21–31 (26.8 ± 3.11) wide at base. Dorsal ray bifurcated near middle or posterior third (n = 9) 11–26 (17.9 ± 5.67) from base, representing (n = 9) 47–78% (60.6 ± 13%) of dorsal ray length; bifurcation with a single papilla, each of two branches containing two pedunculate papillae, disposed on postero-ventral margin. Spicules equal, symmetrical, yellowish brown (n = 12) 105–148 (126.8 ± 12.62) in length; prominent paired alae arising in anterior third (determined from capitulum) extending to near distal spicule tip; alae strongly trabeculate through most of length, with trabeculae becoming indistinct distally. Shaft of spicule not split, tip blunt rounded at extremity, claw-like at lateral-view. Gubernaculum lacking capitulum, composed of bifurcate corpus with paired legs and paired denticulate plates of crura. Corpus thin, arched, elongate (n = 11) 60–86 (73.4 ± 6.95) in total length; composed unpaired anterior portion (body) (n = 11) 32–57 (42.1 ± 6.66) long, bifurcating in two lateral legs (n = 11) 23–36 (31.3 ± 4.27) long, near mid-length; distal tips of legs of gubernaculum situated slightly ventral and medial between denticulate plates of crura. Crurae plates (n = 11) 15–25 (19.5 ± 2.91) long, each with 4–5 denticulate processes (usually five), often not equally distributed in individual specimens; axis of plates slightly twisted. Denticulate crurae with delicate wing-like expansions extending anteriad from proximal end; appear joined ventrally (across corpus split/legs) by relatively narrow hyaline band of tissue. Post-cloacal papilliform protuberances situated antero-ventral to cloaca, disposed ventrally to base of dorsal ray. Telamon present, bar-like at lateral view and poorly developed.

**Females:** Based on specimens from muskoxen (allotype, three additional entire females, and cephalic or caudal extremities). Total length (n = 3) 18.4–21.3 mm (19.4 ± 1.63); maximum width (n = 4) 108–195 (147.3 ± 36.09). Esophagus (n = 4) 265–332 (313.8 ± 28.89) long and 39–64 (49.8 ± 9.04) wide, (n = 3) 1.2–1.8% (1.6 ± 0.32) of body length. Nerve-ring (n = 3) 63–156 (118.7 ± 49.14), cervical papillae (n = 2) 189–217, excretory pore (n = 2) 154–237 from cephalic extremity. Uteri paired, prodelphic; sphincter at termination of uterine limbs (n = 4) 52–57 (54.5 ± 2.89) long. Vagina uterina voluminous, (n = 4) 260–598 (468.8 ± 154.39) long, extending posteriad from sphincter (n = 4) 52–57 (54.5 ± 2.89), vagina vera (n = 4) 62–117 (99 ± 25.26) in length. Vulvar aperture on solid knob-like protuberance; body width at vulva (n = 7) 57–78 (68.4 ± 11.86). Provagina reduced with hood-like fold extending from anterior lip of vulva ventrally across prominent genital protuberance. Perivulval pores situated bilaterally at level of vulva and genital protuberance. Anus in middle to distal third between vulva and tail tip; distance vulva-anus (n = 7) 99–166 (131.3 ± 22.38); vulva-tail (n = 7) 143–215 (178.6 ± 24.76). Tail conical (n = 8) 39–55 (46.3 ± 6.39) long, with lateral phasmids near apex.

*Ova*: Eggs, as determined in lung washes from caribou UC-178-2, spherical to ovoid with delicate, smooth shell (n = 20) 60–78 (68.4 ± 5.39) long by 57–74 (64.9 ± 5.06) wide.

*First-stage larvae*: Body slender, often coiled in life, with paired lateral alae extending from near cephalic extremity to near anus. Tail composed of three segments defined by prominent folds; dorsal spine at level of insertion of the proximal tail fold; tail tip with acutely pointed terminal spike. Meristic data provided in Kutz’s original findings [12], and compared in Table five of [20].

*Second-stage larvae*: Transitional larval stage, non-diagnostic, characterized by variation in developmental attributes relative to age of infection in intermediate host. Usually cuticle of L1 retained; interior of body often appearing vacuolated.

*Third-stage larvae*: Based on 21 fully developed L3 recovered from digested *D. laeve*. Total length (n = 21) 453–540 (497 ± 25.95). Cephalic extremity with papillae surrounding oral aperture. Buccal cavity with prominent, paired stylet-like structures (n = 5) 7.5–8.5 (7.9 ± 0.42). Esophagus claviform (n = 20) 151–210 (178 ± 14.12) long and 14–21 (16.4 ± 2.14) wide, with bulbous formation in anterior; (n = 20) 30–42% (35.9 ± 3.1) of body length. Body width at esophageal base (n = 20) 23–40 (29.8 ± 4.5). Nerve-ring (n = 20) 71–94 (83.8 ± 5.66) in anterior half of esophagus, excretory pore (n = 19) 92–119 (105.5 ± 6.6), and genital primordium (n = 7) 288–400 (349.4 ± 46.7), from cephalic extremity. Tail (n = 21) 25–34 (29.4 ± 3.5) in length, with spike-like protuberance located ventrally on tip (n = 21) 2–5 (3 ± 0.88), structurally variable, ranging from bluntly rounded to slightly elongate and acute.

Additional data based on four fully developed L3 recovered from digested *D. reticulatum*. Total length (n = 4) 451–541 (491 ± 37.34). Esophagus (n = 4) 163–187 (180.3 ± 11.53) long and 15–18 (16.3 ± 1.26) wide, (n = 4) 35–38% (36.8 ± 1.5) of body length. Body width at oesophageal base (n = 4) 31–34 (31.8 ± 1.5). Nerve-ring (n = 2) 72–85 (78.5 ± 9.19), excretory pore (n = 4) 90–108 (101.8 ± 8.5), and genital primordium (n = 3) 301–384 (336.6 ± 42.85), from cephalic extremity. Tail (n = 4) 26–31 (29.8 ± 2.5) in length, with spike-like protuberance located ventrally on tip (n = 4) 2.5–4 (3.3 ± 0.61).

### Taxonomic summary

***Type-host:*** Muskox, *Ovibos moschatus wardi* (Lyddeker, 1900).

***Additional hosts:*** Woodland caribou, *Rangifer tarandus caribou* (Gmelin, 1788). Also known in *Rangifer tarandus groenlandicus* (Borowski, 1780), *Rangifer tarandus grantii* (Allen, 1902), *Alces americanus gigas* Miller 1899, and *Ovibos moschatus moschatus* (Zimmermann, 1780) (in part based on sequence comparisons from Kutz’s original findings [12]).

***Intermediate hosts:*** Natural gastropod intermediate host: *Deroceras laeve* (Müller, 1774) [12]. Experimental intermediate host: *Deroceras reticulatum* (Müller, 1774).

***Predilection site:*** Adult males and females occur in terminal bronchioles and alveoli of lungs based on recovery of few intact worms from repeated tracheal washes, and dissection of minute bronchi.

***Type-locality:*** Tasiujaq, Nunavik Region, Quebec, Canada (58°44′51″N, 70° 02′ 06″W).

***Additional localities:*** Near Tasiujaq and type locality (58° 44′ 10″N; 69° 34′ 18″W); near Kuujjuaq, Nunavik Region, Quebec (58° 45′ 00″N; 68° 33′29″W); and the Cold Lake Air Weapons Range, Alberta, Canada (55° 2′ 25″N; 110° 34′5″W) (type series). Also known to be widely distributed across high latitudes of North America based on prior sampling of DSL (see Table two in [12]).

***Type-specimens:****Adult specimens*: Holotype male from type-host and locality on 7 April 2010 collected by G. Verocai, S. Kutz and M. Simard, USNPC 103740 (GenBank JQ478646). Allotype female on 8 April 2010 collected by G. Verocai, S. Kutz and M. Simard from *O. m. wardi* near type-locality adjacent to Tasiujaq, Quebec, USNPC 103741. Paratypes include males and females in host Om-10-2007 (1 vial), USNPC 103743 collected by M. Simard on 21 March 2007 at Kuujjuaq, Quebec; Om-02-2010 (9 vials), USNPC 103742 (GenBank JQ478649), 103748 (GenBank JQ478647), and 103749 (GenBank JQ478648); and Om-10-2010 (7 vials), USNPC 103744 (GenBank JQ478644-5), collected by G. Verocai, S. Kutz and M. Simard in *O. m. wardi* in Kuujjuaq in animals from Tasiujaq and Kuujjuaq (Table 1), respectively, Nunavik Region, Quebec, on April 7th and 8th , 2010. Additional paratypes from woodland caribou (UC178-2) from the Cold Lake herd, Alberta, found road killed in November 23rd 2010 (Bob McClymont, Alberta FWD), collected by G. Verocai on October, 2011, USNPC 105697 (GenBank JX115006), 105698, and 105699 (GenBank J115007). *First-stage larvae*: Paratypes in Om-02-2010 under accession number USNPC 103742. Additional paratypes used for DSL morphometric and molecular data in [12] are available under the following accession numbers: *O. m. wardi*, Nunavik, QC, USNPC 98648–98652 (GenBank EU018464, 018465, 018479); and *R. t. granti*, North Alaska Peninsula, AK, USNPC 10585, 10586, and 10589 (GenBank EU018478). *Third-stage larvae*: L3 paratypes recovered from experimentally infected terrestrial slug *D. reticulatum*, in September 2008 by J. Ouellet, USNPC 103745 (matching DSL: USNPC103747), and March 2010 by G. Verocai, USNPC 103746 (GenBank JQ478650); and experimentally infected terrestrial slug *D. laeve* by G. Verocai in September 2011, USNPC 107784.

***Etymology:*** The specific name, “*eleguneniensis”* denotes the extensive geographic distribution across northern North America for this protostrongylid. The derivation is from the North Slavey language spoken by the Dene people from the Sahtu Settlement Area, NT, Canada, in which “elegu” means cold and “nene” means land. The term “elegunene” is used by them to refer to the northern lands. This specific name is in recognition of over a decade of collaboration with the Sahtu Dene supporting wildlife health and parasitology research in northern Canada [40].

### Differential diagnosis

In the context of our studies, we accept the validity of the Varestrongylinae and placement of the genus *Varestrongylus* within this restricted group of protostrongylids see [1,2]. Following the characterization of *V. eleguneniensis* the genus now contains ten valid species of lungworms typical of caprine bovids and cervids across the Holarctic [1,12,18-20]. Species are mainly diagnosed by the structure of the copulatory bursa and its rays, and configuration of the spicules and gubernaculum (corpus and plate-like paired crurae) in males and by the structure of provagina in females [1,16,17]. *Varestrongylus eleguneniensi*s is established based on an integration of comparative morphological and molecular criteria for adults and larval parasites.

*Adult nematodes*: Consistent with the current generic diagnosis, males of *V. eleguneniensis* possess prominent, paired denticulate plates of the crurae disposed slightly lateral, dorsal and distal to the legs of the gubernaculum, and a configuration of bursal rays typical to the genus. In females there is a reduced provagina.

*Males*: Among males (Table 2), *V. eleguneniensis* is immediately distinguished by the dimensions of its miniscule spicules from: *V. alpenae*; *Varestrongylus capricola* Sarwar, 1944; *Varestrongylus longispiculatus* Liu, 1989; *V. pneumonicus*; *Varestrongylus qinghaiensis* Liu, 1984; *V. sagittatus*; and *Varestrongylus tuvae* (Boev & Sulimov, 1963); we refer to these seven species as ‘long-spicule forms’ [20]. In contrast, spicules of *V. eleguneniensis* are small (105–148 μm) compared to *>* 200 μm for all of the above species. Further, the diminutive, split gubernaculum (60–86 μm) of *V. eleguneniensis* contrasts with the solid, rod-like corpus typical of *V. capricola*, *V. longispiculatus*, *V. pneumonicus* and *V. tuvae*. The split gubernaculum in other long-spicule forms of *Varestrongylus* (*V. alpenae*, *V. qinghaiensis* and *V. sagittatus)*, has legs that are usually fused by a poorly cuticularized membrane. *Varestrongylus eleguneniensis* is distinguished from all others by the configuration of the denticulate plates of crurae e.g., [1,18,19].

**Table 2:** **Comparative morphometry of males of all valid species within the genus** ***Varestrongylus***

**Species/Characters**	***V. eleguneniensis***			***V. alpenae*** ^**a**^	***V. alces*** ^**b**^	***V. capreoli*** ^**c**^	***V. capricola*** ^**d**^	***V. longispiculatus*** ^**e**^	***V. pneumonicus*** ^**f**^	***V. qinghaiensis*** ^**g**^	***V. sagittatus*** ^**h**^	***V. tuvae*** ^**i**^
	**Range**	**Ex muskox**	**Ex caribou**									
Total length	8.8–14.7	8.8–12.28	11.7–14.7	13–15	11.36–14.7	5.3–13.5	15–18	14.47–17.19	14–24	9–12.2	14.5-33.8	nd
Maximum width	78–147	78–147	84–106	_	68.46–80	32–68	140	62–75	165–200	54–94	112–189	174
Esophagus^§^	247–395	247–377	365–395	230–250	250–272	90–146	275	294–349	310–390	257–338	260-360	_
Esophagus base width	44–62	44–60	52–62	_	32–37	_	50	27–39	_	_	51	_
Body width at esophagus base	70–91	70–91	82	60–65	53.8–61.9	_	_	_	_	46–79	_	_
Nerve-ring^§^	87–196	87–143	182–196	_	68–89.7	_	_	78–99	170–180	_	160	_
Cervical papillae	151–215	151–215	184–200	_	201–207	_	_	_	_	_	_	_
Excretory pore^§^	147–242	147–242	216–234	190–200	208–230.3	_	_	135–189	–	_	240	_
Spicule (right)	105–148	105–140	135–148	375	138.6–163	129–160	250–280	232–282	290–570	80–116.5	325–433.8	361
Spicule (left)	Equal	Equal	Equal	Equal	Equal	Equal	Equal	Sub-equal, 280–314	Equal	Sub-equal, 107.4–160.9	Equal	Equal
Gubernaculum	60–86	60–86	67–72	145–170*	65–83.1	70–86	150	114–147	138–165	104–139	128–176.6	200
Gubernaculum head	Absent	Absent	Absent	Absent	Absent	8–14	Absent	Absent	Absent	Absent	Absent	Absent
Gubernaculum corpus	32–57	32–57	37–42	95–110	38–49	–	_	_	_	_	_	_
Gubernaculum crura	23–36	23–36	30	50–60	24–39.1	–	_	_	_	_	_	_
Crura denticulate piece	15–25	15–25	17–23	>50	15–25	18–30	25	27–29	36–40	27.2–35	33–53.8	NA
Body width at bursa	50–75	50–75	58–65	90–100	42–56	_	_	_	–	49–62	82	nd
Bursa width	95–135	95–135	110–116	350	125–160	_	_	_	165–220	_	_	_
Bursa length	65–91	65–91	80–87	90	75–90	_	_	_	140–165	_	_	_
Dorsal ray length	23–39	23–39	23–36	15	18–30	NA	NA	18–27	NA	NA	NA	NA
Dorsal ray base	21–31	21–31	26–27	10	11.4–15	NA	NA	NA	NA	NA	NA	NA

Total length in millimeters (mm), and all other measurements are in micrometers (μm).

^a^ *V. alpenae*: original description [21]; ^b^ *V*. alces, according to [20]; ^c^ *V. capreoli*: original description [41], plus additional information compiled in [1]; ^d^ *V. capricola*: Measurements from original description cited in [1]; ^e^ *V. longispiculatus*: Measurements from original description [18]; ^f^Data from the original description [42], and additional data from [43] and [1]; ^g^ *V. qinhaiensis*: Measurements from Measurements from [19]; ^h^ *V. sagittatus*: Combined measurements from Measurements from cited in [1,41]; ^i^ *V. tuvae:* Measurements from original description cited in [1]; § Measurements from anterior end; nd = never determined; NA = not applicable.

Specimens of *V. eleguneniensis* are further characterized and distinct from congeners based on a combination of attributes of the male genital system and copulatory bursa. The conformation of the gubernaculum allows recognition of two groups in the genus: (i) species in which the corpus is distally bifurcate with an evident separation of anterior (corpus) and posterior (legs) regions (*V. eleguneniensis*, *V. alces*, *V. alpenae*, *V. capreoli* and *V. qinghaiensis*); or (ii) where the corpus is entire, and usually rod-like, without distal legs (*V. pneumonicus*, *V. sagittatus*, *V. capricola*, *V. tuvae* and *V. longispiculatus*). Further, the distal extremity of the spicules can be either: (i) entire (*V. eleguneniensis*, *V. capreoli*, *V. alces*, *V. longispiculatus* and *V. qinghaiensis*); or (ii) branched (*V. pneumonicus*, *V. sagittatus*, *V. alpenae*, *V. capricola* and*V. tuvae*). In general, in species of *Varestrongylus* spicules are equal in length, except for those in *V. qinghaiensis* and *V. longispiculatus*, whose spicules are sub-equal. Species can also be divided in two groups based on length of the dorsal ray: (i) where the dorsal ray is short, rounded and often indistinct (*V. pneumonicus*, *V. sagittatus*, *V. capreoli*, *V. capricola*, *V. tuvae* and *V. qinghaiensis*); or (ii) where the dorsal ray is elongate with prominent papilliform structures (*V. eleguneniensis*, *V. alces*, *V. alpenae* and *V. longispiculatus*).

Although males of *V. eleguneniensis* are immediately distinguished from seven congeneric species based on spicule length, specimens are most similar to those of *V. alces* and *V. capreoli* in the overall dimensions of the spicules, and structure and dimensions of the gubernaculum. Collectively, these three species characterize the small-spicule forms within the genus. Although equivalent in length, the morphology of the spicule tips and alae effectively distinguish *V. eleguneniensis* from *V. capreoli* and *V. alces*. Spicules are distally entire in the three species, but for *V. eleguneniensis* the paired spicule alae taper distally, are not inflated, and do not reach the apex of the spicule tip; in *V. alces*, a somewhat spatulate condition is apparent distally [20]. The form of the denticulate plates of the crurae is an additional diagnostic feature among these three species. In *V. eleguneniensis* and *V. alces* the relatively “stocky” plates are twisted on the longitudinal axis and have a similar number of denticulate processes, 4–5 in the former and always 5 in the latter; plates in *V. alces* contrast with *V. eleguneniensis* in being strongly arched in dorso-ventral view. In contrast to these species, *V. capreoli* (and specimens identified as *V.* cf. *capreoli*) has strongly triangular plates armed with four acute, prominent teeth. Telamon plates of *V. eleguneniensis* and *V. capreoli* are bar-like and poorly developed, whereas in *V. alces*, these are slightly more developed and triangular in lateral view. The bursa of *V. eleguneniensis* and *V. alces* is dorsally notched with an indistinct dorsal lobe, differing from *V. capreoli,* in which it is weakly bi-lobed. Also, the relative disposition of bursal rays in *V. eleguneniensis* is comparable to that of *V. alces*, but differs from that of *V. capreoli*: the dorsal ray in *V. alces* is slightly elongate and bifurcate instead of short and rounded and the externo-dorsal lateral rays originate independently from each other. Ventral rays in the three species originate from a common stalk, but are distally split in *V. eleguneniensis* and *V. alces*, and basally split in *V. capreoli*.

*Females*: Females of *V. elegunieniensis* differ from *V. capreoli* and *V. alces*, and all other valid species of *Varestrongylus*, by having a strongly reduced provagina (Table 3). The only other species with a reduced provagina is *V. capricola*, although the relative degree of development exceeds that observed in *V. eleguneniensis*. Among other members of *Varestrongylus*, the well-developed provagina (at different degrees) is a membranous, tubular structure extending posterior from the vulva along the ventral aspect of the tail anterior to the anus. Eggs are expelled from the vulva after and are released posterior to the genital protuberance through this series of tubular membranes.

**Table 3:** **Comparative morphometry of females of all valid species within the genus** ***Varestrongylus***

**Characters**	***V. eleguneniensis***	***V. alpenae*** ^**a**^	***V. alces*** ^**b**^	***V. capreoli*** ^**c**^	***V. capricola*** ^**d**^	***V. longispiculatus*** ^**e**^	***V. pneumonicus*** ^**f**^	***V. qinghaiensis*** ^**g**^	***V. sagittatus*** ^**i**^	***V. tuvae*** ^**j**^
Total length	18.41–21.27	20	16.25–21.52	9.4–15	nd	44. 6–51.8	19.6–31	13–18	22–61	>11
Maximum width	108–195	–	73–102	38–95	140	90–120	100–190	64–119	170–300	204
Esophagus^§^	265–337	–	270–310	122–290	400	289–365	360–400	275–320	260–360	–
Esophagus base	39–64	–	30–42	–	65	42–66	–	28.7–37	51	–
Body at esophagus	75–107	–	57–67	–	–	–	–	–	–	–
Nerve-ring^§^	63–156	–	86–97	72–90	–	87–96	190–250	–	160	–
Cervical papillae	189–217	–	150–180	–	–	–	–	–	–	–
Excretory pore^§^	154–237	–	159–220	180–186	–	198–228	–	–	240	–
Tail	39–55	70–75	34–51	34–78	50	66–93	40–60	37–65	90–112	128–149
Vulva-anus	99–166	150–160	70.1–104		–	–	40–60	64–92	–	–
Vulva-tail	143–215	90	108 –146	90–144	–	150–188	80–120	101–157	180–225	255–362
Width at vulva	57–90	100	46–69	–	–	–	–	–	–	162–200
Vagina	377–711	550–600	702–961	–	–	–	–	–	–	–
Eggs length^†^	60–78^†^	60–90	55–67	56–78	65-75	54–63	57–80	86–91	78	–
Eggs width^†^	57–74^†^	25–35	46–63	37–45	30-40	27–30	30–43	12–34	48	–

Total length in millimeters (mm), and all other measurements are in micrometers (μm).

^a^ *V. alpenae*: original description [21]; ^b^ *V*. alces, according to [20]; ^c^ *V. capreoli*: original description [41], plus additional information compiled in [1]; ^d^ *V. capricola*: Measurements from original description cited in [1]; ^e^ *V. longispiculatus*: Measurements from original description [18]; ^f^Data from the original description [42], and additional data from [43] and [1]; ^g^ *V. qinhaiensis*: Measurements from [19]; ^h^ *V. sagittatus*: Combined measurements from cited in [1,41]; ^i^ *V. tuvae:* Measurements from original description cited in [1]; § measurements from anterior end; † eggs collected from lungs of infected caribou, not inside female uteri; nd = never determined.

*Identification of DSL and L3 stages*: Attributes of DSL and L3 stages, but not the L2, were considered because these are the larval stages of diagnostic relevance. We compared morphometric features of both stages to these of other protostrongylids that may occur in a similar spectrum of hosts, or which potentially may be sympatric with *V. eleguneniensis* in North America.

The characteristic dorsal-spine readily separates *V. eleguneniensis* DSL from L1 of species within the Subfamily Protostrongylinae (including known or potentially sympatric *Pr. stilesi*, *Protostrongylus rushi* Dikmans, 1937, and *O. macrotis*), but it is shared among all genera/species within the Subfamilies Elaphostrongylinae and Muelleriinae [2,12]. The morphometry of DSL of *V. eleguneniensis* may overlap not only with other *Varestrongylus* species as previously mentioned, but also with those of other genera within Elaphostrongylinae and Muelleriinae, although larvae of *V. eleguneniesis* are generally shorter than those of the sympatric *Parelaphostrongylus* spp., and *U. pallikuukensis* [3,12,15]. DSL of *V. eleguneniensis* are also shorter than those of *Elaphostrongylus* spp. [3,20,44]. The importance of differentiating these two is uncertain as it is unclear if the new species occurs in sympatry with *E. rangiferi* in Newfoundland, Eastern Canada, where this Eurasian protostrongylid has been introduced [45,46].

The L3 stage of *V. eleguneniensis* appears to be similar to those of species within *Varestrongylus* and Elaphostrongylinae, in which all have a caudal protuberance. Despite this similarity, L3 of elaphostrongylines that geographically co-occur with *V. eleguneniensis (Parelaphostrongylus* spp. and, potentially, *E. rangiferi*) are generally double the size of these of *V. eleguneniensis*, and larval total length may be useful for identification at the generic level (i.e., *Varestrongylus*) (see Table 4; [3,47,48]. L3 of *V. eleguneniensis* can easily be distinguished from those of species within Muelleriinae, in particular the sympatric *U. pallikuukensis*, in which the rounded tail lacks a caudal protuberance [6,35]. Further, L3 of *V. eleguneniensis* appear undistinguishable from those of *V. alpenae*, although the geographic range of this latter is not well characterized, and it is not certain if these congeners may occur in sympatry or are characterized by parapatric ranges in North America. Morphometric data of L3’s should be cautiously interpreted since intra-specific variation exists mainly related to age of larva (i.e., early, intermediate, or late stage, e.g. [36]), but also may reflect variation related to development in different gastropod species.

**Table 4:** **Comparative morphometrics of L3 of** ***Varestrongylus eleguneniensis*** **and selected Protostrongylidae species (Varestrongylinae, Elaphostrongylinae, Muellerinae)**

**Characters**	***V. eleguneniensis*** ^**a1**^	***V. eleguneniensis*** ^**a2**^	***V. alpenae*** ^**b**^	***P. andersoni*** ^**c**^	***P. odocoilei*** ^**c**^	***P.*** ***tenuis*** ^**c**^	***E. rangiferi*** ^**d**^	***U. pallikuukensis*** ^**e**^	***U. pallikuukensis*** ^**f**^
	**(n = 7–21)**	**(n = 4)**	**(n = 18)**	**(n = 10)**	**(n = 10)**	**(n = 10)**	**(n = 15)**	**(n = 10)**	**(n = 29)**
Total length	453–540 (497 ± 25.95)	451–541 (491 ± 37.34)	434-515 (480 ± 20)	911–1,085 (1,003)	738–977 (890)	1,100–1,323 (1,200)	937–1,041 (1,004)	514–600 (560 ± 33.64)	545–691 (648 ± 35)
Esophagus^§^	151–210 (178 ± 14.12)	163–187 (180.3 ± 11.53)	–	322–412 (365)	282–399 (323)	412–521 (463)	338–421 (381)	181–214 (200 ± 11.71)	201–263 (233 ± 13)
Esophagus base width	14–21 (16.4 ± 2.14)	15–18 (16.3 ± 1.26)	–	–	–	–	–	–	18–35 (23 ± 3.1)
Body at esophagus base	23–40 (29.8 ± 4.5)	31–34 (31.8 ± 1.5)	26–30 (28 ± 2)	36–43* (40)	36–52* (44)	47–62* (54)	42–49 (46)	39–60 (47 ± 7)	42–46 (44 ± 1.8)
Nerve-ring^§^	71–94 (83.8 ± 5.66)	72–85 (78.5 ± 9.19)	–	123–130 (128)	135–154 (141)	152–174 (165)	120–150 (139)	93–106 (99 ± 4.23)	83–118 (107 ± 6.7)
Excretory pore^§^	92–119 (105.5 ± 6.6)	90–108 (101.8 ± 8.5)	–	–	–	–	138–163 (153)	109–127 (118 ± 5.01)	104–146 (130 ± 7.9)
Genital primordium^§^	288–400 (349.4 ± 46.7)	301–384 (336.6 ± 42.85)	–	651–738 (697)	521–586 (561)	694–846 (749)	574–648 (615)	318–388 (361 ± 22.74)	316–432 (402 ± 26)
Tail	25–34 (29.4 ± 3.5)	26–31 (29.8 ± 2.5)	–	32–43 (36)	45–50 (47)	48–64 (53)	40–70 (52)	26–34 (31 ± 2.88)	26–34 (31 ± 2.88)
Tail protuberance	2–5 (3 ± 0.88)	2.5–4 (3.3 ± 0.61)	–	–	–	–	–	Not present	Not present

All measurements are given in micrometers (μm).

^a1^Present study, L3 from experimentally infected *Deroceras laeve.* Measurements in variable number of larval specimens (n = 21: Total length, Tail, Tail protuberance; n = 20: Esophagus, Esophagus base width, Body at esophagus base, Nerve-ring; n = 19: Excretory pore; n = 7: Genital primordium);

^a2^Present study, L3 from experimentally infected *Deroceras reticulatum*;

^b^From experimentally infected White-tailed and Mule deer using *Webbhelix multineata* (syn. *Triodopsis multineata)* and *N. albolabris* as IH [26];

^c^From [47] using *W. multineata* as IH. Larval sources: *P tenuis* from White-tailed deer, Rachelwood Wildlife Research Preserve, Pennsylvania; *P. odocoilei* from Mule deer, Jasper National Park, Alberta; *P. andersoni* from White-tailed deer, southeastern BC;

^d^L1 of caribou from Newfoundland (as *Elaphostrongylus cervi*), cited in [12];

^e^From [6] using *D. reticulatum* as IH. Source of L1: muskoxen from Nunavut;

^f^Larvae grown in *D. laeve* as IH. Measurements from ‘late’ and emerged L3 are included [36]. Source of L1: experimentally infected muskoxen [35].

§Measurements from anterior end.

Dashes represent measurements that were not determined, despite of the presence of the character in larvae of the species.

*Body width measured at intersection of esophagus and intestine.

### Emended diagnosis of *Varestrongylus* Bhalerao, 1932

Details from the current study and description of *V. eleguneniensis* along with the recent resurrection of *V. alces* [20] have made it necessary to propose an emended diagnosis for the genus in order to accommodate adequate recognition of some morphological attributes typical of *Varestrongylus.* The need for an emended diagnosis reflects inconsistency in prior descriptions and the names applied to designate some structural features (e.g. [1,20,28,42]). We, hereby, emend the generic diagnosis of *Varestrongylus* as follows:

*Varestrongylus* Bhalerao, 1932 (syn. *Strongylus* Müller*,* 1780 (in part., *sensu* Mueller 1891)); *Protostrongylus* Kamensky, 1905 (in part.); *Synthetocaulus* Railliet & Henry, 1907 (in part.); *Bicaulus* Schulz & Boev, 1940; *Leptostrongylus* Dougherty & Goble, 1946; *Capreocaulus* Schulz & Kadenazy, 1948, *Cystocaulus* Boev, 1950 (in part.).

Metastrongyloidea: Protostrongylidae: Varestrongylinae. Male: dorsal ray of copulatory bursa short or elongate, sometimes almost reduced (*V. capreoli*, *V. tuvae*); apex sometimes bifurcate (*V. alpenae*, *V. longispiculatus*, *V. eleguneniensis*) with variable number of small papillae. Postero-lateral rays of bursa considerably shorter than medio-lateral rays. Telamon plates variable: complex structure (*V. tuvae*), poorly developed, or absent (*V. qinghaiensis*). Spicules of filamentous composition, distally entire or bifurcate, provided with alae and lacking manubrium. Capitulum of gubernaculum present (*V. capreoli sensu stricto*) or absent. Body of gubernaculum in form of long, narrow, and usually colorless structure, entire or distally bifurcate as distinct legs. Paired plates of crurae of gubernaculum (sometimes referred as feet) independent of body or fused by hyaline membrane and in form of two structures with odontoid processes on their edges (denticules) (except in *V. tuvae*, which has smooth feet). Female: provagina always present; reduced (*V. eleguneniensis*, *V. capricola*) or well-developed; tail conical, pointed, often acute. First-stage larva with dorsal spine on tail insertion (DSL), tip of tail kinked and composed by three segments defined by transverse folds. Third-stage larvae possess morphologically variable caudal protuberance. Parasites of lungs in Cervidae: *Cervus* and *Dama* (Cervinae)*,* and *Capreolus, Alces, Rangifer, Odocoileus* (Odocoileinae)*;* and Bovidae (Caprinae): *Ovis, Pseudois*, *Capra*, *Ovibos*, and *Budorcas. Valid species: V. sagittatus* (Mueller, 1890) Dougherty, 1945; *V. pneumonicus* Bhalerao, 1932; *V. alpenae* (Dikmans, 1935) Dougherty, 1945; *V. capreoli* (Stroh & Schmid, 1938) Dougherty, 1945; *V. capricola* Sarwar, 1944; *Varestrongylus alces* Demidova & Naumitscheva, 1953; *V. tuvae* (Boev & Sulimov, 1963) Boev, 1968; *Varestrongylus qinghaiensis* Liu, 1984; *Varestrongylus longispiculatus* Liu, 1989; *V. eleguneniensis* (present study).

## Discussion

*Varestrongylus eleguneniensis* sp. n. is the first protostrongylid ‘true lungworm’ to be described in caribou and is also found in muskoxen and, less often, moose from boreal to Arctic environs of North America, with exception of High Arctic islands of the Canadian Archipelago and Greenland. The description of this species corroborates the discovery of a previously unknown protostrongylid circulating in ungulates across high latitudes of North America [12]. This new taxon is clearly distinct from other protostrongylids, varestrongylines, and species of *Varestrongylus* on the basis of morphological attributes of adult males and females. Molecular sequence data confirm the conspecificity of adult and larval parasites, and provide further differentiation among the small-spicule forms known in the genus [12,15,20]. Current knowledge of geographic distribution and host associations across an extensive range in the northern Nearctic, combined with phylogenetic analysis based on morphological characters (G. Verocai, E.P. Hoberg, unpublished), suggests that caribou (i.e., subspecies of *R. tarandus* native to North America) may be the primary host for *V. eleguneniensis*, as cervids are considered the ancestral hosts for the genus *Varestrongylus* [13].

### Host distribution

*Varestrongylus eleguneniensis* is the only known protostrongylid lungworm associated with subspecies of *Rangifer,* and appears to be restricted to the Nearctic. Previous studies have reported only elaphostrongyline protostrongylids in Eurasian reindeer, e.g. [1,49,50], and subspecies of caribou or introduced semi-domesticated reindeer from North America [1,14,17,45,46,48,51-53]. Prior to the advent of molecular-based diagnostics it is likely that DSL of *V. eleguneniensis* were identified as *P. andersoni* in caribou herds across Canada [12,45,46,48]. These two protostrongylids occur in sympatry and cases of mixed infections have been reported [12,27].

In muskoxen, lung-dwelling protostrongylids were unknown until relatively recently [6,8]. Muskoxen at high latitudes of North America are infected with *U. pallikuukensis* and *Pr. stilesi*, the latter of which is considered to be a result of independent events of host-switching from Dall’s sheep (*Ovis dalli dalli* Nelson) in areas of sympatry in Northwest Territories, Yukon and Alaska ([8]; Verocai, Adams, Kutz, unpublished observations). All confirmed records of *V. eleguneniensis* in muskoxen come from populations sympatric to caribou, either through: (i) long-term sympatry as it occurs in the core of muskox range in mainland central Canadian Arctic [12,15]; (ii) recent translocation events in the 20th century with traceable origins (i.e. Ellesmere Island and Greenland, where protostrongylids have not been detected in caribou nor muskoxen) ([12,27]; present study); or (iii) occur in areas that lungworms until recent years, due to environmental conditions, could not complete their life cycle, and establish [15]. These findings reinforce the hypothesis that this previously unrecognized species has the caribou as its ancestral host. However, given the high prevalence of *V. eleguneniensis* found in some muskox populations sympatric with infected caribou ([12,15]; G. Verocai, M. Simard and S. Kutz, unpublished obs.), we predict that this parasite could be maintained in muskoxen in the absence of caribou.

In contrast to caribou and muskoxen, until now, the Yukon-Alaska moose (*Alces americanus gigas* Miller, 1899) had never been reported as host of protostrongylid lungworms. Pulmonary protostrongylid parasites appear to be rare in other moose subspecies from the Nearctic, although, in southern latitudes, there has been an isolated report of *O. macrotis* in naturally infected *Alces americanus andersoni* Peterson 1952 ([54]; G. Verocai, C. Kashivakura and S. Kutz, unpublished obs.). Moose in Newfoundland are believed to be infected with *P. andersoni* [46,53], along with *E. rangiferi*, but larval identity was not confirmed by molecular techniques and nor were adult worms recovered. Similar to other lungworms, we consider the findings of *V. eleguneniensis* in moose to be relatively isolated and indicative of incidental infections [12].

Available survey data suggest that *V. eleguneniensis* is restricted to the Nearctic, and records for protostrongylids in reindeer, Eurasian moose (*Alces alces* L.), or introduced muskoxen in Eurasia, involve other genera and species. In the Palearctic, only the tissue-dwelling *E. rangiferi* has been recognized in populations of reindeer (*R. t. tarandus* L., *R. t. fennicus* Lönnberg*, R. t. platyrhynchus* Vrolik) [1]. For instance, there have been reports of *E. rangiferi* as far east as Buryatia, near Lake Baikal [50]. Yet, studies on reindeer in the Russian Far East are scarce. The irrefutable veterinary importance of *E. rangiferi* may have led researchers to overlook the potential presence of a small and obscure, pulmonary protostrongylid species such as *V. eleguneniensis* that are associated with significant gross pathology. However, lesions apparently characteristic of infections by *Varestrongylus* sp. have recently been observed in semi-domesticated reindeer (*R. t. tarandus*) from north-central Finland, but the parasite species involved has not been identified (A. Oksanen and S. Laaksonen, Pers. comm., 2010).

The Eurasian moose, congeneric with the North American moose, is recognized as a primary and potentially the only host for *V. alces*, in Russia, Poland, and Fennoscandia [55-61]. Presence of *V. eleguneniensis* in this host is unlikely, as the parasite seems to only incidentally infect moose in Alaska, and has not been found in assessed moose populations in northern Canada [12]. For introduced muskoxen in Norway and Sweden it is also unlikely that *V. eleguneniensis* is present. These animals were originally introduced from Greenland, a location where protostrongylids have not previously been detected in caribou or muskoxen [27]. Dorsal-spined larvae have been reported in muskoxen from Norway and Sweden but are most certainly acquired locally [62], and DSL and adult specimens from the Norwegian population were identified as *M. capillaris* [63]. More extensive field collections are required to completely resolve faunal diversity and host associations for protostrongylids in the northern Palearctic.

### Pathology and significance

*Varestrongylus eleguneniensis* does not appear to cause substantial pulmonary pathology in infected hosts. No gross lesions were observed in any of the muskox and caribou lungs examined in the present study. Also, previously, despite careful examination, our group failed to find lesions in over 50 caribou lungs and over 100 muskoxen lungs from areas that we now know are in the geographic range of *V. eleguneniensis* [12]. Little is known about its pathology and impact on infected ungulates, although light parasitic pneumonia was histologically demonstrated in muskoxen from the same source population (M. Simard, S. Lair, A. Dallaire, Pers. comm.). Further investigations and histological examination of infected lungs are warranted. In contrast, other species of *Varestrongylus*, such as *V. alces* [20,56], *V. capreoli* [41], *V. alpenae* [22,23], and *V. pneumonicus* [43], are known to cause gross lesions, and histopathologic changes.

Co-infections of *V. eleguneniensis* with *P. andersoni* in caribou (G. Verocai, S. Kutz, unpublished data) and *U. pallikuukensis* in muskoxen [15] occur, and could have additive effects on the hosts. Co-infections with other protostrongylids that overlap in host and geographic range, such as *P. odocoilei* in caribou and *Pr. stilesi* in muskoxen have not been reported*.* Additionally, another lungworm, *Dictyocaulus eckerti* Skrjabin, 1931, occurs in caribou and muskox populations across the distribution of *V. eleguneniensis* [12], and at least one muskox evaluated in this study was co-infected (G. Verocai, M. Simard, S. Kutz, unpublished obs.).

### Explorations on historical biogeography

Species of *Varestrongylus* are known from ungulates of the families Cervidae (six species in Cervinae and Odocoileinae (=Capreolinae)) and Bovidae (four species in Caprinae) in Eurasia and North America ([1,20,28]; present study). Eurasia is the center of diversity for both these ungulate groups. The modern tribes of Caprinae and Cervinae originated in Central Asia near 14.7-14.5 Ma (millions of years ago) during the middle Miocene; and Odocoileinae diversified between 11.0-10.0 Ma around the middle and late Miocene boundary [31,64]. Coincidentally, Eurasia is the center of diversity for species within *Varestrongylus,* as well as for other members of Protostrongylidae and a substantially broader strongylate nematode fauna in artiodactyls [6,13,65,66]*.* As a generality, Eurasian biodiversity or species richness among ungulate nematodes considerably exceeds that observed in the Nearctic [13]. Geographically, *Varestrongylus* is characterized by eight species endemic to Eurasia and the western Palearctic [1,20] in contrast to *V. alpenae* and *V. eleguneniensis* which have distributions restricted to North America ([21]; present study).

The formation of the contemporary North American fauna involved expansion from Eurasia, with successive waves of invasion and geographic colonization during the late Pliocene and Quaternary across the Bering Land Bridge [13,64,67-69]. A consequence of these episodic processes has been the development of an extensive faunal mosaic coinciding with asynchronous arrival and recurrent establishment of particular ungulate groups and their associated parasite faunas [13,65,66,70]. During these independent events of geographic colonization, the hosts of the two Nearctic species of *Varestrongylus* entered North America and expanded across much of the continent. The ancestors of *O. virginianus,* the only known host of *V. alpenae*, reached the Nearctic around 4 Ma, whereas the three known hosts of *V. eleguneniensis* invaded and became established in North America in more recent times. Current evidence based on field surveys including collections of adult parasites, fecal examination and sequencing, and studies documenting the distribution of *P. andersoni*, *P. tenuis*, *P. odocoilei*, *Protostrongylus coburni* Dikmans, 1935, *O. macrotis* and *V. alpenae* among species of *Odocoileus* have not revealed the presence of *V. eleguneniensis* in relatively southern host populations [3,10,11,13,21,28].

*Rangifer* is a Beringian endemic, and first arrived to North America approximately 2 Ma, but multiple events of expansion and retraction followed during glacial-interglacial cycles of the Pleistocene, with secondary isolation of *Rangifer* north and south of the Nearctic continental glaciers [71-73]. From Beringia, *Rangifer* also expanded westwards through the Palearctic, resulting in its present Holarctic distribution [71-74]. In contrast, the other two hosts of *V. eleguneniensis* only became established in North America in shallower time. *Ovibos* (as *Ovibos moschatus*) expanded into Beringia around 900–700 Ka (thousands of years ago) and, similarly to *Rangifer,* occurred in isolated populations both in Beringia and environs south of the ice-sheets during the Pleistocene. Currently, natural muskox populations only occur in North America. The species became extinct in the Palearctic in the Holocene [13,64,75]. *Alces*, as a late Pleistocene migrant to the Nearctic, entered the Nearctic only 14–11 Ka, with subsequent eastwards and southwards expansion after the recession of the continental ice [64,76,77].

Historical biogeography of these host-parasite assemblages and development of associations of these parasites with cervids and caprines is complex and can be initially considered in the context of phylogenetic inference among the protostrongylids and species of *Varestrongylus.* An ancestral association with cervids has been proposed for *Varestrongylus* [13], which is supported by the host-associations of the Elaphostrongylinae, the sister group of Varestrongylinae [2], also primarily parasites of cervids [1,3,4,78]. Concurrently, this supports a primary association of *V. eleguneniensis* with caribou and secondary host switching to muskoxen and moose in zones of relatively recent to very recent contact, proposed by Hoberg et al. [13]. This primary association is further supported by the current geographic distribution of *V. eleguneniensis*, which virtually mirrors that of caribou in North America Further, an ancient association with *Rangifer* may indicate that *V. eleguneniensis* may also have been a Beringian endemic during the late Pliocene, but since subsequently multiple expansion events of *Rangifer* have occurred it is impossible to estimate with precision when *V. eleguneniensis* first arrived in the continent [13,68].

A limited phylogenetic analysis (based on ITS-2) among five species of *Varestrongylus* suggests that *V. eleguneniesis* is genetically closer to *V. alces* and *V. capreoli* [20]*.* This putative association of *V. eleguneniensis* with these Eurasian species, as opposed to the only other Nearctic species, *V. alpenae*, appears consistent with at least two independent events of host-parasite invasion from Eurasia, involving Beringia, to the Nearctic during the late Pliocene and Quaternary. The morphological similarities of the two Eurasian species and *V. eleguneniensis*, which collectively form the ‘short-spicule’ group within *Varestrongylus,* further support their relationship (see also [20]). Therefore, we hypothesize that two distinct *Varestrongylus* species crossed Beringia and reached the Nearctic from Eurasia: with *V. alpenae* and *V. eleguneniensis,* or their ancestors, invading North America along with *Odocoileus* and *Rangifer* hosts, respectively. Based on these empirical data, a primary association of *V. eleguneniensis* with muskoxen, a caprine host would not be predicted; its presence in muskoxen is likely the result of several independent host switching events in areas of sympatry with infected caribou, including recent events linked to translocations and introductions (see Host Distribution section above). Also, a primary association with muskoxen would be considerably shallow in time; and perhaps, more difficult considering the strong population bottlenecks and extinctions across its range [75,79]. Contrasting with this distribution, muskoxen are the only recognized hosts for an otherwise relictual protostrongylid species, *U. pallikuukensis* [6,80]. Besides its very recent invasion of the Nearctic, infection of moose with *V. eleguneniensis* is rare and incidental, and is only reported in areas of sympatry with caribou [12].

Considering finer scale geographic and host associations, genetic studies on *Rangifer* distinguish two main lineages of caribou in the Nearctic, the North-American *Rangifer* lineage (NAL), which ranged during glacial maxima south of the Laurentide and Cordilleran ice-sheets, isolated from the Beringian-Eurasian lineage (BEL) vastly distributed from Europe to Beringia [71-74,81].The ancient association of *V. eleguneniensis* with *Rangifer* and this host’s intricate historical biogeography allow us to articulate testable hypotheses on the historical biogeography and phylogeography of this novel lungworm species: (i) the parasite was maintained within BEL caribou and restricted to Beringia, and expanded along with caribou eastwards and southwards, colonizing NAL populations; (ii) the parasite was maintained both within BEL in Beringia and within NAL south of ice sheets, and expanded geographically with both lineages; (iii) the parasite was maintained within NAL, solely south of the ice sheets in one or multiple refugia and expanded northwards with NAL caribou and, later, colonized and expanded with BEL populations. Future studies on the population genetics of *V. eleguneniensis*, involving geographically extensive sampling across its vast range in North America, may reveal genetic signatures compatible to such events of expansion and/or isolation within a single or multiple refugia.

## Conclusions

Herein we have described and named *V. eleguneniensis*, a pulmonary protostrongylid with *Rangifer* as a primary definitive host; that secondarily infects muskoxen and moose in areas of sympatry. The parasite appears to be geographically restricted to North America; however there is a lack of surveys for pulmonary protostrongylids in *Rangifer* from Eurasia, including western Beringia. Detailed investigations for the presence of *V. eleguneniensis*, its close relative *V. alces*, or another *Varestrongylus* in reindeer from the Palearctic remain necessary. The biogeographic history for two endemic species of *Varestrongylus* known from North America appears consistent with events of parasite invasion with cervid hosts from Eurasia into North America during the late Pliocene and Quaternary. The putative ancient association with *Rangifer* hosts could be investigated through the phylogeography of *V. eleguneniensis*, which may provide new insights on caribou historical biogeography and the history of colonization of the Nearctic by host-parasite assemblages.
